# Identification of *Drosophila* Gene Products Required for Phagocytosis of *Candida* *albicans*

**DOI:** 10.1371/journal.pbio.0040004

**Published:** 2005-12-20

**Authors:** Shannon L Stroschein-Stevenson, Edan Foley, Patrick H O'Farrell, Alexander D Johnson

**Affiliations:** **1**Department of Microbiology and Immunology, University of California, San Francisco, California, United States of America; **2**Department of Biochemistry and Biophysics, University of California, San Francisco, California, United States of America; Stanford UniversityUnited States of America

## Abstract

Phagocytosis is a highly conserved aspect of innate immunity. We used *Drosophila melanogaster* S2 cells as a model system to study the phagocytosis of *Candida albicans,* the major fungal pathogen of humans, by screening an RNAi library representing 7,216 fly genes conserved among metazoans. After rescreening the initial genes identified and eliminating certain classes of housekeeping genes, we identified 184 genes required for efficient phagocytosis of *C. albicans*. Diverse biological processes are represented, with actin cytoskeleton regulation, vesicle transport, signaling, and transcriptional regulation being prominent. Secondary screens using *Escherichia coli* and latex beads revealed several genes specific for *C. albicans* phagocytosis. Characterization of one of those gene products, Macroglobulin complement related (Mcr), shows that it is secreted, that it binds specifically to the surface of *C. albicans,* and that it promotes its subsequent phagocytosis. Mcr is closely related to the four *Drosophila* thioester proteins (Teps), and we show that TepII is required for efficient phagocytosis of *E. coli* (but not *C. albicans* or *Staphylococcus aureus*) and that TepIII is required for the efficient phagocytosis of *S. aureus* (but not *C. albicans* or *E. coli*). Thus, this family of fly proteins distinguishes different pathogens for subsequent phagocytosis.

## Abstract 2

Mcr and the closely related *Drosophila* Tep proteins (proteins similar to mammalian secreted immune complement) bind to the surface of invading microbes and are required to promote the phagocytosis of specific pathogens.

## Introduction

Dedicated host phagocytic cells suppress microbial proliferation by engulfing pathogens and subsequently engaging additional host defenses through cytokine production and antigen presentation [1–3]. Pathogen recognition activates signaling pathways within the phagocytic cell that induce the rearrangement of the actin cytoskeleton and thereby lead to engulfment of the pathogen. For example, signaling activates Rho family GTPases, which regulate actin assembly during phagocytosis [4]. In addition to actin rearrangements, insertion of new membrane occurs at the site of phagosome assembly, contributing to the membrane protrusions elaborated during engulfment [5,6]. Although the morphological changes and cytoskeletal rearrangements that occur during phagocytosis have been well described, there have been, until recently, few attempts to comprehensively identify the cellular components that are required for phagocytosis, and these have used bacterial species [7–10]. With this in mind, we have undertaken a systematic approach to identify host genes required for the efficient phagocytosis of a fungal pathogen, *Candida albicans*.

*C. albicans* is a common commensal fungal organism found in the gastrointestinal tract and other tissues of more than 50% of healthy adults [11]. Extremes of age, injury, antibiotic use, and a compromised immune response predispose individuals to the development of mucosal or life-threatening systemic infections. *C. albicans* is the now the fourth most common organism detected in systemic infections [12], and mortality approaches 35% [13]. The predisposition of neutropenic and HIV+ patients with decreased CD4+ T cells to *C. albicans* infections suggests that both innate immunity and acquired cell-mediated immunity are involved in mediating host resistance to *C. albicans* infections [3,14].

The genetically tractable fruit fly, *Drosophila melanogaster,* is a well-established system for studying conserved components of innate immunity [1,2,15]. For example, studies in *Drosophila* were instrumental in revealing the significance of Toll signaling in the innate immune response [16,17]. In addition, *Drosophila* has been successfully used to study the interaction of several human pathogens—including *Listeria monocytogenes, Plasmodia, Mycobacterium marinum,* and *C. albicans*—with conserved features of the innate immune system [18–22].

*Drosophila* plasmatocytes are macrophage-like cells and the predominant of three distinct hemocyte types: they phagocytose cell debris and invading microbes. Phagocytosis appears important for full immunity against pathogens, as blocking phagocytosis in *imd* mutant flies sensitized them to infection with *Escherichia coli* [1]. The *Drosophila* S2 cell line is believed to be derived from embryonic plasmatocytes and shares many properties with plasmatocytes, including robust phagocytosis [23]. The advent of RNA interference (RNAi) and the availability of genomic RNAi libraries open new possibilities for the exploration of phagocytosis in this model system. RNAi in S2 cells has been used to systematically study phagocytosis of *E. coli*, *L. monocytogenes,* and *Mycobacterium fortuitum* [7,9,10], and RNAi in vivo in *Anopheles gambiae* to study phagocytosis of *E. coli* and *Staphylococcus aureus* [8]. In this paper, we describe a high-throughput assay to study the phagocytosis of *C. albicans* by the S2 cell line. Using this RNAi-based screen, we have explored the roles of 7,216 evolutionarily conserved genes in the phagocytosis of *C. albicans* by S2 cells. From this screen, we identified 184 genes required for the efficient phagocytosis of *C. albicans*. In many cases, we identified multiple subunits of known protein complexes or multiple components of known biochemical pathways. In secondary assays, we distinguished genes specifically required for the phagocytosis of *C. albicans* from those required generally for phagocytosis. We performed additional experiments with one such *Candida*-specific component, Macroglobulin complement related (Mcr), a protein highly conserved in metazoans (CG7586). We show that Mcr is required for the efficient phagocytosis of *C. albicans,* but not that of *E. coli, S. aureus,* or latex beads. We also demonstrate that Mcr is secreted into the media of S2 cells and binds the cell surface of *C. albicans,* presumably leading to recognition and phagocytosis by S2 cells. We also show that Mcr exhibits specificity in its recognition of the cell surface of *C. albicans*. Mcr binds more to wild-type *C. albicans* than to a *C. albicans* mutant *(Δefg1/Δefg1)* or to the common laboratory strain of *Saccharomyces cerevisiae* (S288c), both of which are poorly phagocytosed. Mcr has four close relatives in the *Drosophila* genome, thioester proteins (Tep), known as TepI, TepII, TepIII, and TepIV (TepI: CG18096; TepII: CG7052; TepIII: CG7068; TepIV: CG10363). None of the four Teps is required for efficient phagocytosis of *C. albicans;* however, we show that TepII is required for efficient phagocytosis of the gram-negative bacteria *E. coli* and TepIII for efficient phagocytosis of the gram-positive bacteria *S. aureus*. These findings show that different members of this conserved group of five proteins show specificity for different pathogens and support studies conducted in mosquito that suggested that these proteins act as a primitive complement system targeting pathogens for immune destruction [8,24,25].

## Results

### Phagocytosis of *C. albicans* by *Drosophila* S2 Cells

**Figure 1:**
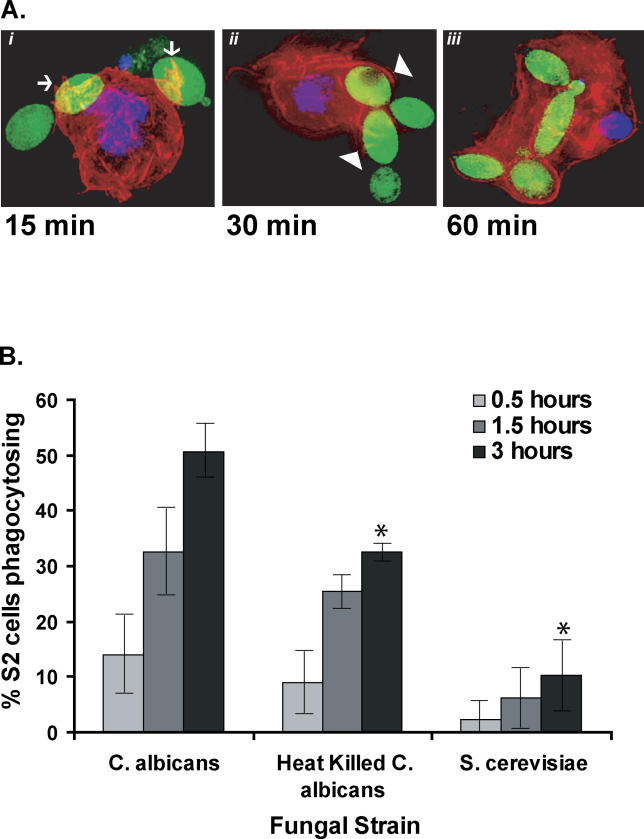
Phagocytosis of *C. albicans* by *Drosophila* S2 Cells (A) The *Drosophila* hemocyte-like S2 cell line phagocytoses *C. albicans*. S2 cells were co-incubated with GFP expressing *C. albicans* for the indicated times. Cells were fixed, and the filamentous actin of S2 cells was stained with rhodamine phalloidin and the S2 cell DNA with Hoechst 33258. (B) Quantification of phagocytosis of *C. albicans* and *S. cerevisiae* by S2 cells. S2 cells and the indicated fungal strain were co-incubated for various times, and the percentage of S2 cells that had phagocytosed one or more *C. albicans* was quantified by counting 50–100 S2 cells. The maximum time shown is 3 h, as the levels of phagocytosis did not significantly increase after this timepoint. Results are the average of four experiments, and the error bars indicate the standard deviation. As described in Materials and Methods, the 3-h results were evaluated for statistical significance using the *t*-test, assuming unequal variance. As indicated by the asterisks, the values for heat-killed *C. albicans* and *S. cerevisiae* were statistically different from that of live *C. albicans,* with a confidence level *p* < 0.01.

*D. melanogaster* is rapidly emerging as a model system to study numerous human pathogens including *C. albicans* [7,9,18–20,22]. To investigate whether the hemocyte-derived fly S2 cell line efficiently phagocytoses *C. albicans,* we co-incubated green fluorescent protein (GFP)–expressing *C. albicans* with S2 cells, fixed the samples, and monitored adherence and phagocytosis of *C. albicans* (Figure 1A). Within a few minutes of mixing, many S2 cells were bound by *C. albicans,* and some S2 cells exhibited actin-rich cytoplasmic protrusions forming toward the *C. albicans* (arrows in panel i in Figure 1A). By 30 min, evidence of *C. albicans* phagocytosis was clear, with the actin cytoskeleton forming pseudopodia engulfing the *C. albicans* (arrowheads in panel ii in Figure 1A). After 1 h of co-incubation, many S2 cells had engulfed at least one *C. albicans* cell (panel iii in Figure 1A). Engulfment of *C. albicans* led to changes in S2 cell morphology, including condensation and displacement of the DNA. To quantify the phagocytosis, we scored the percentage of S2 cells that had internalized one or more *C. albicans* cells (Figure 1B). Under these conditions, approximately 50% of S2 cells phagocytosed at least one *C. albicans* within 3 h. Phagocytosis did not critically depend on the *C. albicans* cells being alive, as heat-killed *C. albicans* cells were phagocytosed in significant amounts (Figure 1B). The difference between live and heat-killed *C. albicans* is probably attributable to the fact that live *C. albicans* were actively dividing over the timecourse, whereas the numbers of heat-killed *C. albicans* obviously did not increase. In contrast to *C. albicans,* a common laboratory strain of *S. cerevisiae* (S288c) was poorly phagocytosed, indicating that there may be a specific mechanism of recognition of *C. albicans* not shared by all fungi. We detected only a low level of adherence of *S. cerevisiae* to the surface of S2 cells, indicating the reduced phagocytosis is likely due, at least in part, to a difference in recognition of *S. cerevisiae* versus that of *C. albicans* (unpublished data).

### Identification of Genes Required for Phagocytosis of *C. albicans*

**Figure 2:**
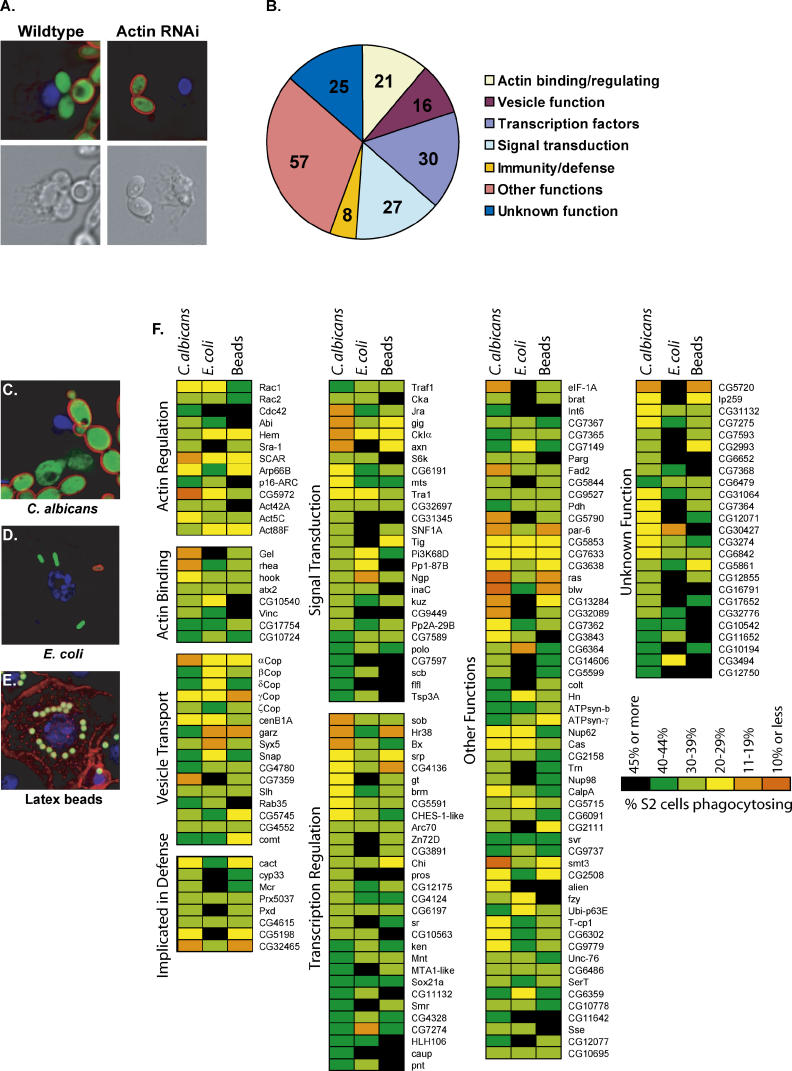
Identification of Genes Required for Phagocytosis of *C. albicans* (A) High-throughput assay for phagocytosis. GFP-expressing *C. albicans* (green) were co-incubated with S2 cells to allow phagocytosis. Cells were lightly fixed, and non-phagocytosed *C. albicans* were secondarily labeled with a rabbit anti–*C. albicans* antibody and Cy3-labeled anti-rabbit antibody (red). S2 cell DNA (blue) was labeled with Hoechst 33258. Left panels, wild-type S2 cells; right panels: S2 cells treated with RNAi against actin (Act5C). (B) One hundred eighty-four dsRNAs decreased phagocytosis of *C. albicans*. The 184 genes were categorized and plotted in a pie graph with the number of genes in each class indicated. (C–E) Secondary screens used to further test the RNAi-treated S2 cells specificity of phagocytosis of *C. albicans* (C), *E. coli* (D), or latex beads (E). (C) Phagocytosis of *C. albicans*. GFP-expressing *C. albicans* (green) were co-incubated with S2 cells to allow phagocytosis. Cells were lightly fixed, and non-phagocytosed *C. albicans* were secondarily labeled with an anti–*C. albicans* antibody and Cy3-labeled anti-rabbit antibody (red). S2 cell DNA was labeled with Hoechst 33258 to mark the position of the S2 cell. The level of phagocytosis was quantified by counting the percentage of S2 cells that had phagocytosed one or more *C. albicans*. (D) *Drosophila* S2 cells phagocytose *E. coli*. GFP-expressing *E. coli* (green) were co-incubated with S2 cells to allow phagocytosis in a similar assay as (C). Cells were lightly fixed, and an anti–*E. coli* antibody was used to label non-phagocytosed *E. coli* (red). The level of phagocytosis was quantified by counting the percentage of S2 cells that had phagocytosed one or more *E. coli*. (E) *Drosophila* S2 cells phagocytose 2-μm latex beads. Yellow-green fluorescent latex beads were co-incubated with S2 cells to allow phagocytosis. The S2 cell filamentous actin cytoskeleton was labeled with rhodamine phalloidin (red) and the DNA with Hoechst 33258 (blue). The level of phagocytosis was quantified by counting the percentage of S2 cells that had phagocytosed one or more latex beads. (F) One hundred eighty-four dsRNAs disrupt the phagocytosis of *C. albicans* by S2 cells. The genes required for phagocytosis of *C. albicans* are listed along with the effect on phagocytosis of *E. coli* and latex beads. The color-based scale is given below and corresponds to the percentage of S2 cells that phagocytosed one or more *C. albicans, E. coli,* or latex beads. Genes were categorized based upon function as in (B). Mean values for phagocytosis by wild-type, untreated S2 cells were: *C. albicans* 52%, *E. coli* 56%, and latex beads 51%.

As phagocytosis is such a critical element of metazoan immune responses to invading microorganisms, we used a library of 7,216 double-stranded RNAs (dsRNAs) representing most of the phylogenetically conserved genes of *D. melanogaster* to identify cellular components responsible for phagocytosis of *C. albicans*. In an initial visual screen, S2 cells treated with dsRNA for 4 d were mixed with GFP-expressing *C. albicans*. Cells were lightly fixed without detergent to prevent permeabilization of the plasma membrane and stained with a polyclonal antibody against whole-cell lysates of *C. albicans* and a Cy3 conjugated secondary antibody. Under these conditions, *C. albicans* cells in the media fluoresce green with a red outline, while those phagocytosed by S2 cells appear only green (Figure 2A). Wells were visually scored for a reduction in the number of phagocytosed cells, visible as green-only cells (Figure 2A). The effect of each of the 7,216 dsRNAs was independently scored by two investigators. The small numbers of discrepancies in the initial pass were resolved through additional screening. In addition, wells with reduced phagocytosis were further investigated to rule out a general cytotoxic effect for a decrease in phagocytosis, as described in Materials and Methods. In all cases, the scoring was carried out without knowledge of the genes represented by the dsRNA.

From this screen, we initially identified 401 dsRNAs that significantly decreased S2 cell phagocytosis of *C. albicans*. A significant number of these genes represented different subunits of large protein assemblies that carry out general aspects of gene expression and protein turnover in the cell. For example, we identified 45 different ribosomal subunits, 31 RNA processing enzymes, 15 general transcription proteins, and 30 proteasome subunits (Table S1). The high frequency of dsRNAs in these categories is not due to lack of specificity of the approach. Indeed, the fact that we identified so many of the ribosome and proteasome subunits indicates a high degree of internal consistency. We reasoned that genes involved in these generic processes are likely to influence phagocytosis indirectly and hence have excluded them from further analysis. We note that another screen for phagocytosis of intracellular bacteria obtained and excluded a large class of ribosome and proteasome genes [7].

We resynthesized and retested the remaining 280 dsRNAs for reduction of *C. albicans* phagocytosis. For this analysis, we quantified the efficiency of phagocytosis and rejected dsRNAs whose effects failed to meet a specified criterion. The levels of *C. albicans* phagocytosis were quantified as the percentage of S2 cells that had phagocytosed one or more *C. albicans,* based on a sample size of roughly 100 S2 cells for each dsRNA. We defined a significant decrease in phagocytosis as anything less than 1.5 standard deviations below the mean for untreated cells (52%). Thus, dsRNAs that reduced the percentage of S2 cells phagocytosing *C. albicans* to 44% or below were scored as statistically significant as explained in Materials and Methods. One hundred eighty-four of the dsRNAs passed this additional test, which was more rigorous than our initial screen (Figure 2B); thus, 96 genes, many of which were probably false positives, were eliminated in the secondary screen. This new set of 184 genes includes 52 genes known from prior studies [4,6,7,9,26–28] to function in phagocytosis and 132 genes whose roles in phagocytosis have not been previously described. Of the genes previously known to function in phagocytosis, Rac1, Rac2, Cdc42, other regulators of actin dynamics, actin itself, all five CopI vesicle proteins, Syx5a (t-Snare), Snap, PI3K, and InaC (Protein Kinase C) were identified in the screen, thereby confirming the validity of the methodologies (Figure 2 and Table S2). Because the dsRNA collection represented conserved genes, many of the biochemical functions of the genes not previously implicated in pathogenesis can be surmised. The 184 genes in the screen can be broken down into the following categories: (1) actin, actin-regulating, and actin-binding proteins (21 genes); (2) vesicle transport, including all five CopI vesicle coat proteins (Cop), several snare proteins, and several regulators of vesicle function (16 genes); (3) sequence-specific DNA-binding proteins that presumably regulate gene transcription (30 genes); (4) signaling components, seven of which have been previously implicated in phagocytosis (27 genes); (5) a set of genes annotated as being involved in immunity and defense against pathogens (eight genes); (6) a catchall category of miscellaneous functions, including protein degradation, metabolism, protein transport, and protein folding (57 genes); and (7) conserved genes having no known function (25 genes).

Agaisse et al. and Philips et al. [7,9] described genes required for the microbial entry and survival in S2 cells of two bacteria, *M. fortuitum* and *L. monocytogenes,* by S2 cells, and it is useful to compare the results of these screens with our results for *C. albicans*. Of the 184 genes identified in our screen, only 21 and 33 genes, respectively, are shared with these two other screens. Most of the overlapping genes encode actin regulatory proteins and vesicle transport proteins, suggesting these processes are particularly important for phagocytosis. There are several possible reasons why the overlap among the screens is not more extensive, and they are taken up in the Discussion.

Of the genes identified in our screen, we wished to distinguish those specifically required for phagocytosis of *C. albicans* from those with a more general role in phagocytosis. We therefore tested all 184 dsRNAs that impaired *C. albicans* phagocytosis for their effects on the phagocytosis of GFP-expressing *E. coli* and yellow-green fluorescently labeled latex beads (Figure 2C–2E). Wild-type S2 cells efficiently phagocytose *E. coli* (Figure 2D) and, to a much lesser extent, latex beads (Figure 2E). The phagocytosis of latex beads required much longer incubation times to detect significant amounts of phagocytosis, an observation that presumably reflects the lack of pathogen-specific signals on their surface. While most dsRNAs identified in the screen affected phagocytosis of all three challengers, a small number affected phagocytosis of *C. albicans* only. Other dsRNAs reduced phagocytosis of all three challengers but affected *C. albicans* to a much greater extent than that of *E. coli* or latex beads (Figure 2F; see Table S2). For the remainder of this study, we concentrate on one dsRNA that specifically impaired phagocytosis of *C. albicans*. This RNA corresponds to a gene annotated in Flybase as *Drosophila* Mcr.

### Mcr-Dependent Phagocytosis of *C. albicans*

**Figure 3:**
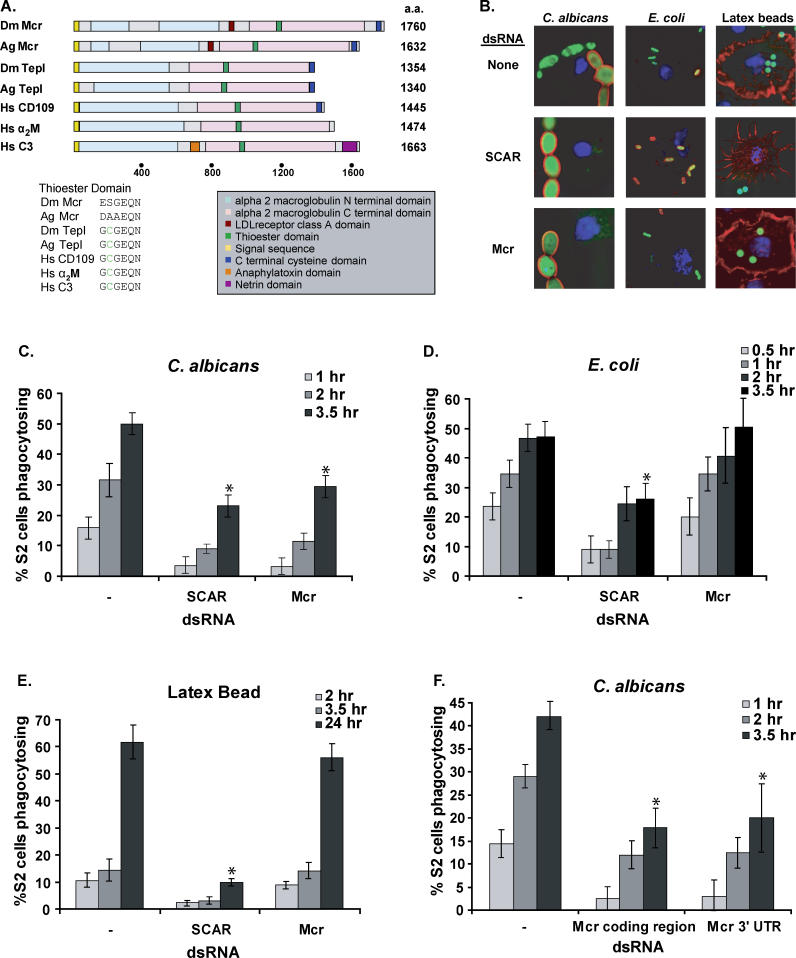
Mcr-Dependent Phagocytosis of *C. albicans* (A) Schematic representation of α2M-related proteins. *Drosophila* Mcr is compared with a close homolog in *A. gambiae* (Ag Mcr [Tep13]), TepI from both *Drosophila* and *Anopheles* and the human homologs CD109, α2M, and C3. Various conserved domains are colored as indicated in the gray box. Numbers correspond to amino acid position. The sequences of the conserved thioester domains are given below the schematic. Dm, *Drosophila melanogaster;* Ag, *Anopheles gambiae;* Hs, *Homo sapiens*. (B) RNAi against SCAR reduces phagocytosis of *C. albicans, E. coli,* and latex beads (row 2). RNAi against Mcr significantly decreased phagocytosis of only *C. albicans* (row 3). Cells were stained as in Figure 4C–4E. Column 1, GFP expressing *C. albicans*—green, S2 cell DNA—blue, non-phagocytosed *C. albicans*—red; column 2, GFP-expressing *E. coli*—green, S2 cell DNA—blue, non-phagocytosed *E. coli*—red; column 3, latex beads—green, S2 cell DNA—blue, S2 cell actin cytoskeleton—red. (C) dsRNA against both SCAR and Mcr decreases phagocytosis of *C. albicans*. S2 cells were treated with dsRNA against SCAR and Mcr as described in Materials and Methods and then co-incubated with *C. albicans* for the indicated times. The percentage of S2 cells phagocytosing one or more *C. albicans* was quantified and plotted. The 3.5-h timepoints were analyzed using a *t*-test assuming unequal variance. Those values that differ significantly from untreated cells (*p* < 0.01) are indicated by asterisks. (D) Mcr dsRNA does not reduce phagocytosis of *E. coli*. S2 cells were treated with dsRNA against SCAR and Mcr and then co-incubated with *E. coli* for the indicated times. The percentage of S2 cells phagocytosing one or more *E. coli* was quantified and plotted. The 3.5-h timepoints were analyzed using a *t*-test assuming unequal variance. Those values that differ significantly from untreated cells (*p* < 0.01) are indicated by asterisks. (E) Mcr dsRNA does not reduce phagocytosis of latex beads. S2 cells were treated with dsRNA against SCAR and Mcr and then co-incubated with green fluorescent latex beads for the indicated times. The percentage of S2 cells phagocytosing one or more latex beads was quantified and plotted. The 3.5-h timepoints were analyzed using a *t*-test assuming unequal variance. Those values that differ significantly from untreated cells (*p* < 0.01) are indicated by asterisks. (F) An additional dsRNA against the 3′ UTR of Mcr was generated and tested for disruption of *C. albicans* phagocytosis. S2 cells were treated with RNAi directed against both the coding region and the 3′ UTR of Mcr and then co-incubated with *C. albicans* for the indicated times. The percentage of S2 cells phagocytosing one or more *C. albicans* was quantified and plotted. The 3.5-h timepoints were analyzed using a *t*-test assuming unequal variance. Those values that differ significantly from untreated cells (*p* < 0.01) are indicated by asterisks.

The *Drosophila* Mcr protein is a member of the α2Macroglobulin/complement family of proteins (Figure 3A). To confirm its selectivity for *C. albicans,* we quantified with a detailed timecourse the relative phagocytosis of *C. albicans, E. coli,* and latex beads by S2 cells treated with dsRNA against Mcr or, as a control, against SCAR dsRNA (Figure 3B–3E). SCAR is an actin-nucleating protein that is required for the phagocytosis of *C. albicans, E. coli,* and latex beads (see Table S2). RNAi against Mcr reduced phagocytosis of *C. albicans,* but not *E. coli* or latex beads when compared with untreated cells. In contrast, RNAi against SCAR strongly affected all three reactions (Figure 3B–3E). To confirm that the reduction in phagocytosis after treatment with Mcr dsRNA was indeed due to a reduction in the Mcr gene product, we designed a second dsRNA against the 3′ untranslated region (UTR) of Mcr. RNAi against either Mcr or the Mcr 3′ UTR similarly reduced phagocytosis of *C. albicans,* confirming that Mcr is indeed important for phagocytosis of *C. albicans* (Figure 3F).

### The Mcr/Tep Family of Proteins Determine Specificity of Pathogen Phagocytosis by *Drosophila* S2 Cells

**Figure 4:**
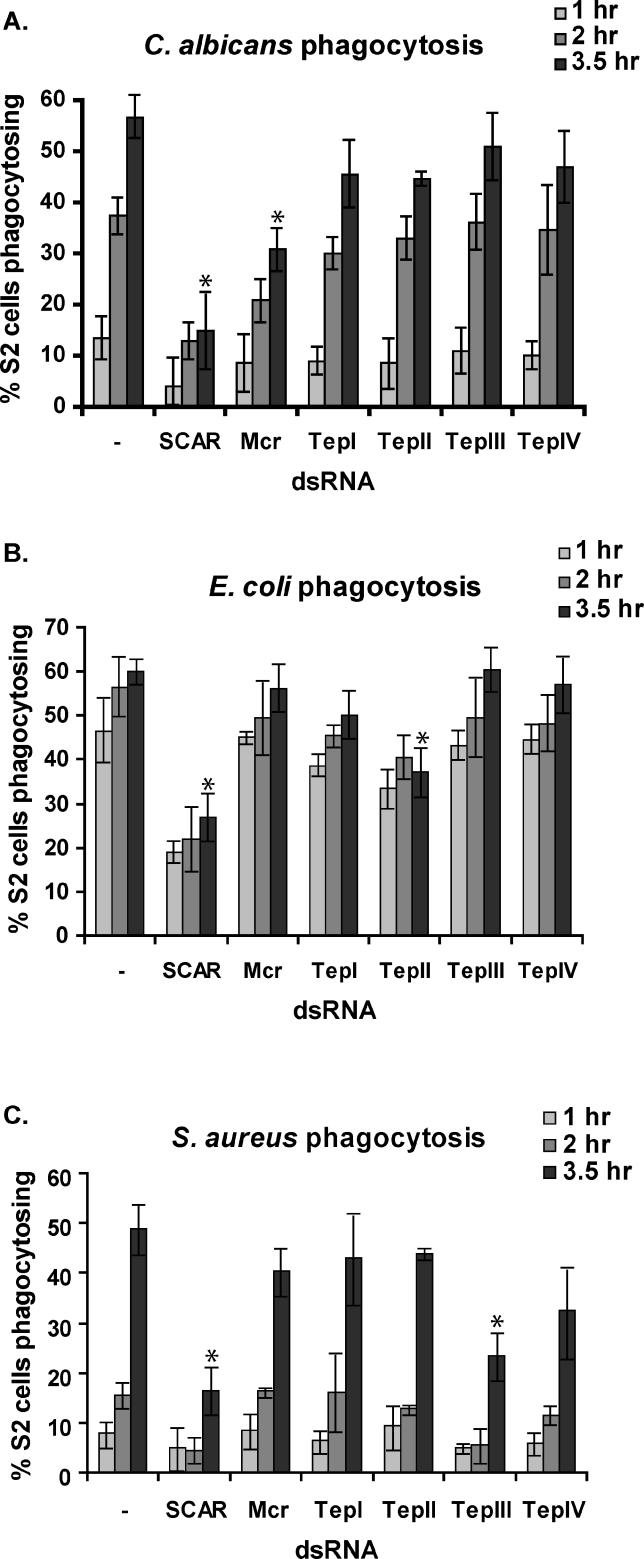
The Mcr/Tep Family of Proteins Determine Specificity of Pathogen Phagocytosis by *Drosophila* S2 Cells (A) S2 cells were treated with dsRNA against SCAR, Mcr, or one of the *Drosophila* Teps and co-incubated with *C. albicans*. The percentage of S2 cells phagocytosing one or more *C. albicans* was quantified and plotted. (B) The S2 cells treated above were also co-incubated with *E. coli,* and phagocytosis was quantified. (C) The RNAi-treated S2 cells above were co-incubated with *S. aureus,* and phagocytosis was quantified. In all graphs, the 3.5-h timepoints were analyzed using a *t*-test assuming unequal variance. Those values that differ significantly from untreated cells (*p* < 0.01) are indicated by asterisks.

Mcr is closely related to a family of four Teps in *Drosophila* [29]. Indeed, Mcr has been referred to in at least one publication as Tep6 [30]; however, it lacks the cysteine residue that forms the defining thioester of the Teps. TepI, TepII, TepIII, and TepIV were represented in our library of dsRNAs, yet Mcr was the only family member identified in the screen as being required for phagocytosis of *C. albicans*. This finding and characterization of mosquito Tep1 suggest that Mcr and the four Teps may be involved in the phagocytosis of specific classes of pathogens [8,25]. To test this idea, S2 cells treated with dsRNA against SCAR, Mcr, and each of the four Teps were tested for their ability to phagocytose three different pathogens (Figure 4). To represent a broad spectrum of pathogens, we used *C. albicans, E. coli* (a gram-negative bacterium), and *S. aureus* (a gram-positive bacterium). RNAi against SCAR reduced phagocytosis of all three pathogens (Figure 4A–4C). Of Mcr and the Teps, phagocytosis of *C. albicans* was only decreased by dsRNA against Mcr (Figure 4A), an outcome predicted from the results of the screen. Phagocytosis of *E. coli* was decreased only by TepII dsRNA (Figure 4B), and *S. aureus* by TepIII dsRNA (Figure 4C). These results indicate that Mcr and the four Teps constitute a family of proteins, the members of which provide specificity in the phagocytosis of different pathogens.

### S2 Cells Secrete Mcr into the Culture Medium

**Figure 5:**
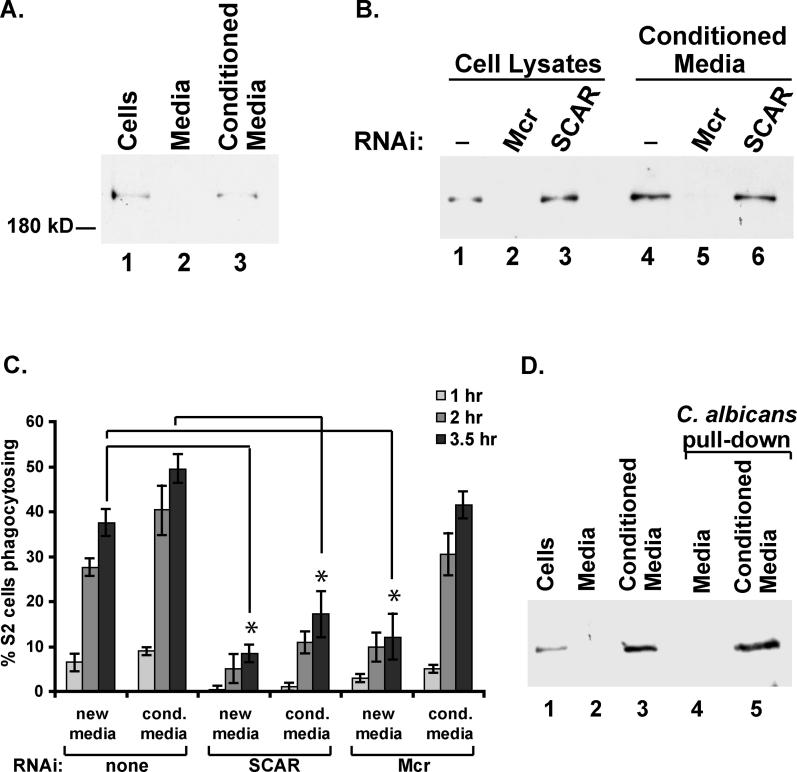
S2 Cells Secrete Mcr into the Culture Media (A) Mcr is secreted into the culture media. Whole-cell lysates were prepared from S2 cells (lane 1) and compared to Schneider's medium with 2% FBS (lane 2) or Schneider's medium with 2% FBS collected from S2 cells (conditioned media, lane 3) by immunoblotting with an anti-Mcr antibody. (B) RNAi against Mcr depletes Mcr protein from cell lysates and from the conditioned media. Cell lysates and conditioned media were collected from wild-type S2 cells or cells treated with RNAi against Mcr or SCAR and probed by immunoblotting with an anti-Mcr antibody. (C) Conditioned media rescues the phagocytosis defect of Mcr RNAi-treated cells. Wild-type S2 cells or cells treated with RNAi against Mcr or SCAR were plated in new Schneider's medium with 10% FBS or conditioned media with 10% FBS from wild-type S2 cells and incubated with *C. albicans* for various times. The percentage of S2 cells that had phagocytosed one or more *C. albicans* was quantified and graphed. A *t*-test was used to test the statistical significance between wild-type cells in new media versus SCAR- or Mcr RNAi–treated cells in new media and wild-type cells in conditioned media versus SCAR- or Mcr RNAi–treated cells in conditioned media (see Materials and Methods). An asterisk indicates comparisons that showed statistically significant differences (*p* < 0.01). Mcr RNAi–treated cells in wild-type-conditioned media were not significantly different from wild-type cells in conditioned media. (D) Mcr interacts with *C. albicans* cells. *C. albicans* was co-incubated either with new media containing 2% FBS or conditioned media containing 2% FBS from wild-type S2 cells for 2 h, washed, and analyzed by immunoblotting with anti-Mcr. Lane 1, S2 cell lysates; lane 2, new media; lane 3, conditioned media; lane 4, *C. albicans* incubated in new media; lane 5, *C. albicans* incubated in conditioned media.

It seems likely that Mcr is a secreted protein, since it contains a putative signal sequence and is related to mammalian α2M, to mammalian complement components, and to *Anopheles* aTep1, all of which are known to be secreted (see Figure 4). Cell lysates and conditioned media from S2 cells were analyzed by SDS-PAGE and Western blotting with a primary antibody raised against a peptide located near the amino terminus of Mcr (Figure 5A). Full-length Mcr (approximately 200 kD) was detected in both cell lysates and in conditioned media, indicating that Mcr is a secreted protein. To verify that this 200-kD species is Mcr, we analyzed its levels in cells and media that were treated with RNAi (Figure 5B). RNAi against SCAR did not significantly change the levels of Mcr in either cell lysate or conditioned medium, whereas RNAi against Mcr significantly reduced the levels of the 200-kD protein in both of them (Figure 5B). If Mcr secretion is relevant to phagocytosis, providing Mcr in conditioned media should alleviate the phagocytosis defect in S2 cells treated with RNAi against Mcr. At the start of the phagocytosis assay, untreated S2 cells, SCAR RNAi-treated S2 cells, and Mcr RNAi-treated S2 cells were diluted into either fresh media or conditioned media (from untreated S2 cells) and tested for phagocytosis of *C. albicans* (Figure 5C). SCAR RNAi and Mcr RNAi both severely suppressed phagocytosis in fresh media. The addition of conditioned media slightly increased phagocytosis by SCAR-treated cells but significantly increased phagocytosis by Mcr-treated cells to levels near those of untreated cells. Conditioned media from Mcr RNAi–treated cells only partially increased phagocytosis activity, indicating that conditioned media from wild-type S2 cells is required for a full rescue of phagocytosis (unpublished data). These results indicate that Mcr presence in the conditioned media is required to rescue the loss of phagocytosis by Mcr RNAi treatment (Figure 5C). These experiments also indicate that Mcr is efficiently synthesized and secreted by S2 cells prior to their exposure to *C. albicans*.

### Specific Binding of Mcr to the *C. albicans* Cell Surface

**Figure 6:**
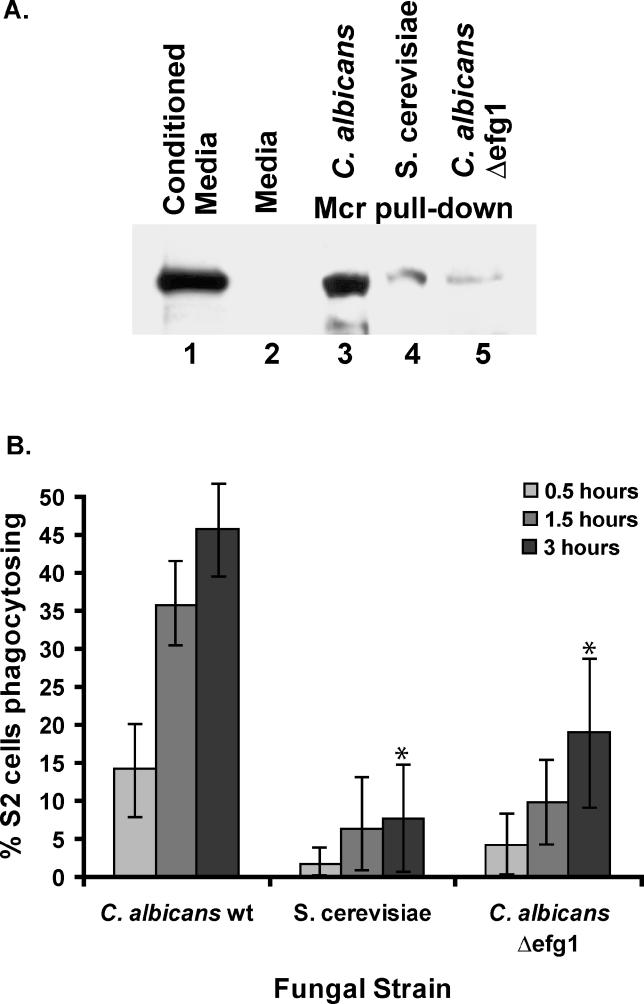
Specific Binding of Mcr to the *C. albicans* Cell Surface (A) Wild-type *C. albicans, S. cerevisiae,* or *Δefg1/Δefg1* mutant *C. albicans* were co-incubated with conditioned media containing 2% FBS from wild-type S2 cells for 2 h, washed, and analyzed by immunoblotting with anti-Mcr antibody. Lane 1, conditioned media; lane 2, new media; lane 3, Mcr bound to wild-type *C. albicans*; lane 4, Mcr bound to *S. cerevisiae*; lane 5, Mcr bound to *Δefg1/Δefg1* mutant *C. albicans*. (B) Quantification of phagocytosis of *C. albicans* wild-type and mutant strains and *S. cerevisiae* by S2 cells. S2 cells and the indicated fungal strain were co-incubated for various times, and the percentage of S2 cells that had phagocytosed one or more *C. albicans* was quantified by counting 50–100 S2 cells. Results are the average of four experiments, and the error bars indicate the standard deviation. The 3.5-h timepoints were analyzed using a *t*-test assuming unequal variance. Those values that differ significantly from untreated cells (*p* < 0.01) are indicated by asterisks.

To test whether Mcr can directly bind to the surface of *C. albicans* cells, *C. albicans* cells were incubated with conditioned media from untreated S2 cells, precipitated, washed extensively, and analyzed by Western blotting using the Mcr antibody. As shown in Figure 5D, the Mcr in the conditioned media efficiently bound to *C. albicans* and remained bound during the extensive washing steps. The binding of Mcr to *C. albicans* is specific, as demonstrated by the following two experiments. First, the binding of Mcr shows a marked preference for *C. albicans* (strain Caf2–1) over the common *S. cerevisiae* lab strain S288C (Figure 6A). This observation parallels the more efficient phagocytosis of *C. albicans* Caf2–1 compared with *S. cerevisiae* S288c (see Figure 1B and Figure 6B). Second, the binding of Mcr shows a preference for wild-type *C. albicans* over a mutant of *C. albicans* deleted for the EFG1 gene, *Δefg1/Δefg1* (see Figure 6A). EFG1 encodes a transcriptional regulator that regulates many genes, some of which affect properties of the cell wall [31,32]. Although we do not know the precise defects in the *Δefg1/Δefg1* strain, the fact that Mcr binds more poorly to it than to a wild-type strain argues that Mcr binding must be specific for some feature on the *C. albicans* cell surface. As predicted from this idea, we found that the *Δefg1/Δefg1* mutant is poorly phagocytosed by S2 cells (see Figure 6B). These experiments, taken together, support the idea that Mcr recognizes features of the *C. albicans* cell surface and that binding of Mcr to *C. albicans* results in its efficient phagocytosis by S2 cells.

## Discussion

Phagocytosis of invading pathogens is a critical component of metazoan innate immune systems. In this study, we investigate the phagocytosis of *C. albicans,* the most prevalent fungal pathogen of humans, using *Drosophila* as a model host organism. *Drosophila* has been well established as a model system for analyzing human microbial pathogens [18–22].

### Identification of Genes Important for Phagocytosis of *C. albicans* by Fly S2 Cells

Although the morphological features and cytoskeletal rearrangements underlying phagocytosis are well described, there have been few comprehensive attempts to identify the core requirements for phagocytosis. None of these have investigated phagocytosis of a fungal pathogen [7–10,33]. Using a dsRNA library representing 7,216 *Drosophila* genes conserved in other metazoans, we carried out an RNAi-based screen to identify genes required for efficient phagocytosis of *C. albicans* by S2 cells. Following rescreening and after eliminating genes encoding ribosomal subunits, proteasome subunits, general transcription factors, mRNA processing enzymes, and other components involved in general aspects of gene expression and protein turnover, we identified 184 genes required for the efficient phagocytosis of *C. albicans* by S2 cells. Among genes well known to function in phagocytosis, we identified actin itself, many of its regulators including SCAR, and multiple components of the Arp2/3 complex [4]. Phagocytosis involves extensive rearrangements of the cytoskeleton, and the identification of these genes in the screen was strongly predicted from prior work [4,7,9]. We also identified all five Cops in the CopI vesicle coat and several SNARE proteins. CopI vesicles are thought to be indirectly required for pseudopod extension because they are required for the maintenance of a pool of VAMP3 (vSNARE)–containing endomembrane vesicles. These vesicles are thought to be needed for insertion into the plasma membrane during pseudopod formation and phagocytosis [6]. The extent of the increase in surface area is dramatized by the large increase in size of S2 cells when phagocytosing several *Candida* (see Figure 1A). We also identified a number of previously implicated signaling components, including PI3K, which is required for both insertion of exocytic membranes in the plasma membrane [27,28] and phagosome maturation [26].

It is important to note that although our screen identified the majority of genes previously implicated in phagocytosis it did not identify all of them. For example, although three subunits of the Arp2/3 complex were identified in the screen, four were not, even though their corresponding dsRNAs were present in our library. There are several possible explanations for this type of failure, including poor RNAi, potentially lethal effects of certain RNAs, and the timing of RNAi treatment. For example, in the case of profilin, the timing of RNAi treatment was critical for detecting its role in cytokinesis [34]. Thus, genes present in the library but not identified in our screen can be rigorously excluded from having a role in phagocytosis of *C. albicans* only through additional experimentation. In any case, the fact that the screen, which was carried out blindly with respect to the identities of the dsRNAs, identified many known components of phagocytosis confirms the validity of the approach. It also implicates the large number of remaining genes as having important roles in this process. All 184 genes identified in the screen are summarized in Table S2. Taken as a whole, these genes represent a number of different cellular processes, including cytoskeletal rearrangements (21 genes), vesicle transport (16 genes), signal transduction (27 genes), and transcriptional regulation (30 genes) (see Results and Tables S1 and S2). We also identified three looser categories of genes: a set of genes encoding diverse functions in metabolism, protein turnover, and transport (57 genes); a set of genes annotated in Flybase as implicated in defense (eight genes); and a set of genes of unknown function, despite their being conserved among metazoans (25 genes).

**Figure 7:**
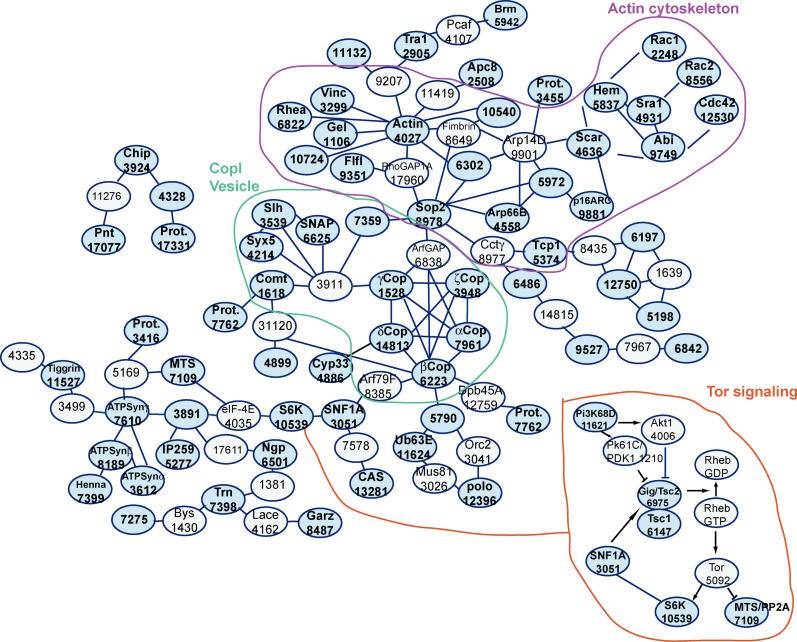
Genes Identified in the Screen as Being Required for Phagocytosis of *C. albicans* Were Superimposed onto a *Drosophila* Genomic Yeast Two-Hybrid Interaction Map Interactions are displayed, and several pathways are outlined. Dark-blue circles indicate genes identified as reduced phagocytosis of *C. albicans,* with light-blue circles indicating genes present in the two-hybrid map but not identified in the phagocytosis screen. Several functional groups are circled as indicated. This diagram represents only a portion of the complete two-hybrid map [35], indicating that the genes identified in the phagocytosis screen affect a limited number of cellular processes.

Giot et al. [35] described a partial interaction map of *Drosophila* proteins, based largely on two-hybrid experiments. If the genes identified by our screen are superimposed on this map (keeping in mind that, because the interaction map is incomplete, many genes are simply not represented in the map), they form a highly interactive network that covers only a portion of the total map space (Figure 7). Many of the newly identified components can be seen to interact directly or indirectly with a known component of phagocytosis. From this analysis, many testable predictions can be made regarding the roles of specific gene products identified in the phagocytosis screen. For example, an unstudied transmembrane protein with predicted Ser/Thr kinase activity (CG5790) interacts with βCop and therefore may be directly involved in vesicle transport during phagocytosis or may be critically regulated by vesicle transport. A second example covers the target of rapamycin (TOR) kinase signaling pathway. Although *Drosophila* TOR itself was not represented in our library, our RNAi screen identified many pathway members, including PI3K, SNF1A (AMPK), S6K, TSC1, Gigas (TSC2), InaC (PKC), and MTS (PP2A) (see Figure 7). This pathway includes two TOR complexes, one responding to growth factors, nutrients, and energy (ATP) and leading to changes in cell size and number and the other regulating actin organization by an unknown mechanism [36]. Our results suggest that phagocytosis is closely connected to the TOR pathway and could impinge upon this unknown mechanism. Many additional insights and predictions regarding the role of individual gene products in phagocytosis can be gleaned from this type of analysis. We view the list of 184 genes implicated in phagocytosis as a resource that will stimulate future approaches and discoveries.

In evaluating the results of any RNAi screen, it is of interest to compare them with analogous screens. Two 2005 analyses examined the ability of an S2 cell line to support intracellular infection by *M. fortuitum* and *L. monocytogenes* [7,9]. Of the 184 genes identified in our screen, 21 genes were shared with the *M. fortuitum* screen and 33 with the *L. monocytogenes* screen. The overlapping genes encoded key components of actin dynamics and vesicle transport; as described above, both processes are well known to have critical roles in the uptake of pathogens and inert particles, and perhaps these two groups of genes define the core processes of phagocytosis. The lack of a more extensive overlap might be due to certain technical differences in the screens (different RNAi libraries, different screening protocols, or different significance thresholds, etc.). However, we believe that much of the difference is due to the nature of the pathogen investigated. *M. fortuitum* and *L. monocytogenes* are intracellular bacterial pathogens, whereas *C. albicans* is a fungal pathogen that is not believed to proliferate intracellularly. Indeed, genes whose role we have characterized in the most detail (Mcr and Tep genes) were not uncovered in these other screens, and we failed to detect the CR gene that was the focus of one of these reports [9]. These results suggest that important features of the recognition or phagocytosis process may be relevant to some pathogens, but not others. Reciprocally, it should be emphasized that not all of the genes scored are likely to be directly involved in phagocytosis. For example, as noted by Ramet [10], the Serpent transcription factor, which was detected as required for phagocytosis in the screen for *E. coli* phagocytosis and our screen, is required for differentiation of hematopoietic cells and appears to be defective in phagocytosis because of a general change in cellular phenotype. By directing our attention to genes that are pathogen specific, we hope to favor the identification of genes directly involved in the recognition process. We are particularly interested in the eight gene products identified in our screen and annotated in Flybase as being involved in the defense response, as they represent a variety of different types of proteins, none of which had previously been shown to be required for phagocytosis, and none of which were identified in the *M. fortuitum* and *L. monocytogenes* screens [7,9]. Several of these genes have established roles in immunity, including an IκB homolog, Cactus, two peroxidases involved in reactive oxygen metabolism, and Cyp33, which is expressed in T cells and regulates gene expression. In other cases, the annotation of these genes was based simply upon the expression pattern of homologous genes in other organisms. For example, the mammalian homolog of CG4615 is expressed in macrophages but not in monocytes. Two of these eight genes, Mcr and Cyp33, appear to be specifically required for the phagocytosis of *C. albicans* when compared with *E. coli*.

### Mcr and Related Proteins

An original aim of our work was to understand how a particular pathogen, *C. albicans,* is efficiently phagocytosed by fly S2 cells. As described, we tested which of the 184 genes identified in this study were specific for *C. albicans* and which were required more generally for phagocytosis. To this end, we rescreened the 184 dsRNAs for effects on the phagocytosis of *E. coli* and latex beads. Although *C. albicans* and *E. coli* are taken up much more rapidly than beads, phagocytosis of beads could be reproducibly measured over longer incubation times. Most of the 184 dsRNAs inhibited phagocytosis generally, although several had a much greater effect on *C. albicans* (see Figure 2). We chose one of these *C. albicans*–specific genes, Mcr, for further analysis.

Mcr is a member of the α2 macroglobulin/complement family (see Figure 3A), which includes at least 11 family members in humans. Members include secreted protease inhibitors (α2M, PZP) and components of complement (C3, C4A, C4B, and C5). Several other family members (CD109, CPAMD8, Ovostatin 1, Ovostatin 2) have fewer well-characterized functions. The prototypical member, α2 macroglobulin (α2M), binds to secreted proteases, including those of pathogens, leading to their uptake and inactivation by host cells. Human α2M also interacts with cytokines to regulate their distribution and activity [37]. The complement cascade is an ancient response to pathogens that triggers opsonization of a pathogen, formation of a membrane-attack complex, and in vertebrates the activation of the adaptive immune system [38]. Historically, the complement cascade was thought to reside only in vertebrates; however, studies suggest its presence in lower eukaryotes, including ascidians and sea urchins [39], and studies in mosquito documented immune functions of the complement-related Teps [8,24,25].

*Drosophila* encodes five proteins that are closely related to the human α2M/complement family of proteins. These are Mcr, the component identified in our screen, and also TepI, TepII, TepIII, and TepIV. The human protein most closely related to the Mcr-Tep family is CD109, a GPI-anchored protein whose function has not been fully explored [40,41]. Teps are expressed in *Drosophila* larvae and adult flies upon infection by *E. coli* [29,42]. The function of the Teps is probably best understood in the mosquito *A. gambiae*. *A. gambiae* Tep1 (aTep1) is required for phagocytosis of *E. coli* by a mosquito cell line [25]. aTep1 is secreted, proteolytically processed, and a fragment adheres to *E. coli* through formation of a thioester bond. During infections of adult mosquitoes by *Plasmodium berghei* parasites, aTep1 binds the surface of the parasite. RNAi knockdown of aTep1 allows more parasite oocysts to survive in the midgut of the mosquito, suggesting that aTep1 plays a critical role in killing malarial parasites [24]. This aTep1-dependent killing of malarial parasites occurs in a compartment devoid of hemocytes and does not involve phagocytosis. The findings suggest that aTep1 may target a microbe to multiple immune defense pathways [24]. The Tep1 thioester bond is presumed to form at a conserved thioester domain (GCGEQN) that is found in all four *Drosophila* Teps, aTep1, human CD109, and other members of the human α2M/complement class of proteins. Mcr and a close relative in *A. gambiae* lack the critical cysteine through which these covalent bonds are formed.

In this study, we show that Mcr is secreted by S2 cells and binds tightly to *C. albicans* in the absence of S2 cells. The defect in phagocytosis caused by Mcr RNAi can be reversed through the addition of conditioned media from normal S2 cells, suggesting that secreted Mcr may be the active Mcr required for efficient phagocytosis of *C. albicans*. Unlike aTep1 or complement in mammals [25,38,39], we did not detect any evidence of proteolytic processing of Mcr, suggesting that the full-length protein is the active form. This may be related to the fact that Mcr lacks the critical cysteine residue present in the Teps and presumably does not form thioester linkages. Mcr appears specific for the phagocytosis of *C. albicans,* as its reduction by RNAi had little or no effect on phagocytosis of *E. coli* or *S. aureus*. Moreover, Mcr binding exhibits specific recognition for *C. albicans* compared to even closely related fungi. Thus, Mcr binding shows a marked preference for *C. albicans* over the common *S. cerevisiae* lab strain S288c. In addition, Mcr binds significantly more to wild-type *C. albicans* than to a *C. albicans* mutant *(Δefg1/Δefg1)* that has altered cell-wall properties. Both *S. cerevisiae* and the *C. albicans Δefg1/Δefg1* mutant are poorly phagocytosed by S2 cells, further supporting the idea that Mcr plays a critical role in recognizing wild-type *C. albicans* and promoting its subsequent phagocytosis.

Given that Mcr appears specific for *C. albicans* phagocytosis, we also investigated the possible roles of the four closely related *Drosophila* Teps. RNAi directed against TepII specifically reduced phagocytosis of *E. coli,* a gram-negative bacterium, and RNAi directed against TepIII specifically reduced phagocytosis of *S. aureus,* a gram-positive bacterium. None of the Tep reductions had any effect on phagocytosis of *C. albicans.* Thus this family of five closely related proteins collectively functions to promote the phagocytosis of a diverse set of pathogens, with individual family members showing specificity for certain classes of pathogens.

Unbiased screens for the genes required for specific pathogen recognition should give a broad view of the mechanisms targeting innate immune responses. Our studies implicate Mcr as one important contributor to the recognition of *C. albicans*. This result is supported by other findings suggesting that the related Teps have a similar role. The Teps have been shown to be related to complement, and in the mosquito *A. gambiae*, Tep1 is required for the phagocytosis of *E. coli* by mosquito 5.1* cells and killing of the malaria parasite in vivo [24,25]. Our analysis of the different *Drosophila* Teps show that they too are specialized in the recognition of different pathogens. But what of the other genes showing *C. albicans*–specific effects? Much remains to be done to determine whether recognition is combinatorial with different genes contributing to different branches of the recognition, or whether, like the complement system of mammals, recognition by Mcr is complex, involving multiple components in the recognition and the signaling.

## Materials and Methods

### Strains and plasmids

The *C. albicans* CAF2–1 strain (URA3/*ura3::λimm434*) was used for most experiments [43]. GFP–*C. albicans* expresses GFP under the control of the ADH1 promoter [44]. The deletion strain *Δefg1 (Δura3::λimm434/Δura3::λimm434 Δefg1::hisG-URA3-hisG/Δefg1::hisG)* has been described previously [45]. The *S. cerevisiae* wild-type strain used was MATα S288c [46]. GFP–*E. coli* (DH5α) expresses GFP under the control of the bacterial ribosomal promoter [47,48]. Shirley Lowe (University of California, San Francisco, United States) kindly provided *S. aureus*.

### Cell culture

*Drosophila* S2 cells were cultured in Schneider's medium (Invitrogen, Carlsbad, California, United States) supplemented with 10% fetal bovine serum (FBS), penicillin, and streptomycin (pen/strep).

### RNAi

The dsRNA library used in this screen has been described previously [48]. S2 cells were plated into 96-well plates at a density of 50,000 cells per well in a culture volume of 150 μl per well. dsRNA was added to a final concentration of 10 μg/ml, and the cells were incubated for four days at 25 °C to allow depletion of the corresponding gene product.

### Phagocytosis screen

Primary screen: 1 × 10^5^ dsRNA-treated S2 cells were plated in 96-well plastic tissue culture plates in 150 μl of Schneider's medium with 10% FBS and pen/strep. FITC-labeled (VWR, West Chester, Pennsylvania, United States, 5mg/ml), GFP-expressing *C. albicans* was added to each well containing S2 cells at a density of 2 × 10^5^ *C. albicans* per well and incubated for 2 h at 25 °C. S2 and *C. albicans* mixtures were transferred to glass-bottom, Concanavalin A–coated 96-well microplates (Greiner Bio-One, Longwood, Florida, United States) and incubated for 1 h. Cells were fixed and processed as described below. Wells were visually screened for an apparent decrease in phagocytosed *C. albicans* (green-only) cells. Both SLS and EF independently screened all wells without knowledge of the identity of the dsRNAs. The few discrepancies between SLS and EF screens were resolved with further analysis. In wells with fewer phagocytosed *C. albicans,* further examination of the S2 cells was performed to rule out cytotoxic effects. dsRNAs were eliminated from further analysis if there were no remaining S2 cells. In wells with fewer S2 cells than normal, the well was examined further for phagocytosis by the remaining S2 cells. In many of these wells, the remaining S2 cells still phagocytosed *C. albicans* normally, and those were not scored as having a phagocytosis defect.

Secondary screens with *C. albicans*: the 280 positive dsRNAs were resynthesized and rescreened with more rigorous standards by visually quantifying the number of S2 cells phagocytosing. Briefly, 1 × 10^5^ dsRNA-treated S2 cells were plated in 96-well plastic tissue culture plates in 150 μl of Schneider's medium with 10% FBS and pen/strep. FITC-labeled (VWR, 5mg/ml), GFP-expressing *C. albicans* was added to each well containing S2 cells at a density of 5 × 10^5^ per well and incubated for 2 h at 25 °C. S2 and *C. albicans* mixtures were transferred to glass-bottom, Concanavalin A–coated 96-well microplates (Greiner) and incubated for 1 h. Phagocytosis assays were performed using similar conditions for DH5α *E. coli* expressing GFP (5μl of an overnight saturated culture per well, phagocytosis for 2 h at 25 °C), and yellow-green fluorescently labeled 2-μm latex beads (Sigma, St. Louis, Missouri, United States, 2 × 10^6^ beads per well, phagocytosis for 20 h at 25 °C). Cells were fixed and processed as described below. While the overall efficiency of phagocytosis varied between experiments, phagocytosis by wild-type S2 cells was highly consistent within each experiment. For this reason, all secondary screen phagocytosis assays were completed in 1 d with the same batch of S2 cells. S2 cells were counted and scored for having phagocytosed one or more *C. albicans, E. coli,* or latex beads. As described in the Results, we used a significance threshold of 1.5 standard deviations below the mean for these secondary screens. A *t*-test was used to compare the percentage of phagocytosis of six wild-type wells to a value of 44% for the dsRNA-treated wells. The significance values (*C. albicans p* = 0.012, *E. coli p* = 0.001, latex beads *p* = 0.005) indicated that these criteria are reasonably stringent, and 44% represents a statistically significant threshold.

### Phagocytosis assays

dsRNA-treated S2 cells (1 × 10^5^) were plated in 96-well plastic tissue culture plates in 150 μl of Schneider's medium with 10% FBS and pen/strep. GFP-expressing *C. albicans* was added to each well containing S2 cells at a density of 5 × 10^5^ *C. albicans* per well and incubated for various times at 25 °C. S2 and *C. albicans* mixtures were transferred to glass-bottom, Concanavalin A–coated, 96-well microplates (Greiner) and incubated for 1 h. Phagocytosis assays were performed using similar conditions for DH5α *E. coli* expressing GFP (5 μl of an overnight saturated culture per well), *S. aureus* (30 μl of a FITC-labeled overnight culture per well), or yellow-green fluorescently labeled 2-μm latex beads (Sigma), 1 × 10^6^ beads per well. Cells were fixed and processed as described below. S2 cells were counted and scored for having phagocytosed one or more *C. albicans* or other pathogen. All graphs represent the mean ± the standard deviation of at least four counted samples. The difference between wild-type and RNAi treatments was statistically analyzed by *t*-test assuming unequal variances. A *p*-value < 0.01 was considered significant.

### Immunofluorescence and microscopy

After co-incubation, the cell culture media was aspirated and allowed to dry for 2 min. Cells were fixed with 1% formaldehyde in PBS for 5 min, washed with 1× PBS, and blocked with 5% FBS in PBS for 2–4 h. The cell surfaces of *C. albicans, E.coli,* or *S. aureus* were detected with primary antibodies raised against whole *C. albicans* (Biodesign, Saco, Maine, United States, Cat# B65411R), whole *E. coli* (Biodesign, Cat# B47711G), or whole *S. aureus* (Biodesign, Cat# B65881R). Primary antibodies were visualized with Cy3-conjugated goat anti-rabbit or rabbit anti-goat antibodies (Jackson ImmunoResearch, West Grove, Pennsylvania, United States). In each case, pathogens were considered phagocytosed if they were not visualized with their corresponding antibody. DNA was visualized with Hoechst 33258, and filamentous actin was detected with rhodamine-coupled phalloidin (both from Molecular Probes, Eugene, Oregon, United States). Immunofluorescent images were taken with a Zeiss Axiovert 200M microscope using AxioVision software (Carl Zeiss, Oberkochen, Germany). Images were processed using Zeiss AxioVision 3D Deconvolution software, and figures were assembled with Adobe Photoshop and Illustrator (Adobe Systems, San Jose, California, United States).

### Western blotting

S2 cells were harvested as described [48], and conditioned media were collected from cells grown for 48 h. Cell lysate and conditioned media were separated by SDS-PAGE and analyzed by Western blotting. Mcr was detected by using a polyclonal rabbit antibody generated against a peptide in the amino half of Mcr (CGQTNPSDRPPYRTDSGS) (Bethyl Laboratories, Montgomery, Texas, United States). To detect Mcr interactions with *C. albicans,* 5 × 10^7^ *C. albicans,* mutant strain or *S. cerevisiae,* were incubated in 3 ml of new Schneider's medium with 2% FBS, or in conditioned media with 2% FBS, for 2 h. *C. albicans* cells were washed one time with media and one time with wash buffer (0.05% NP-40, 120 mM NaCl, 50 mM Hepes [pH 7.5], and 5 mM EDTA) and loaded on SDS-PAGE gel with SDS-sample buffer and analyzed by Western blotting with the Mcr antibody.

## Supporting Information

Table S1 Genes Not Further Followed Up with Secondary Assays dsRNAs that decrease the phagocytosis of *C. albicans* but were not followed up in further studies, including genes involved in general transcription, translation, RNA processing, and the proteasome.
(207 KB DOC).

Table S2 Measurement of the Percentage of S2 Cells Phagocytosing after Treatment with dsRNA against Individual Genes dsRNAs that decrease the phagocytosis of *C. albicans.* Column 3, closest human homolog; column 4, function of *Drosophila* protein if known, function is based upon mammalian or other homologs if *Drosophila* protein function unknown; column 5, genes that have previously been implicated in phagocytosis; columns 6–8, the percentage of S2 cells that have phagocytosed one or more *C. albicans* (C.a.), *E. coli* (E.c.), or latex beads (beads). 50–100 S2 cells were counted for each condition.
(679 KB DOC).

### Accession Numbers

The FlyBase (http://flybase.bio.indiana.edu/search/) accession numbers for the genes and gene products discussed in this paper are Mcr (FBgn0020240), TepI (FBgn0041183), TepII (FBgn0041182), TepIII (FBgn0041181), and TepIV (FBgn0041180). The NCBI Entrez (http://www.ncbi.nlm.nih.gov/gquery/gquery.fcgi) accession number for *A. gambiae* Ag Mcr (Tep13) is EAA12257.2.

## Abbreviations

Cop: coat protein
dsRNA: double-stranded RNA
GFP: green fluorescent protein
Mcr: Macroglobulin complement related
RNA interference: RNAi
Tep: thioester protein
TOR: target of rapamycin
UTR: untranslated region
