# Supplementary material for: Identification of Drosophila Gene Products Required for Phagocytosis of Candida albicans
Source: PLoS Biol. 2005 Dec 20;4(1):e4. doi: 10.1371/journal.pbio.0040004 (PMC1310651; doi:10.1371/journal.pbio.0040004)
Supplement: Supplementary file 1 [file pbio.0040004.st001.doc]

Table S1. Genes not further followed up with secondary assays

| Name | CG | Function |  |
| --- | --- | --- | --- |
| RpII215; HDAC3; RpII140; Cdk9; His3.3A; CG6121; CycT; Med21; dalao; Spt5; CG11265; Rpb5; Spt6 nej; Trf2; RpL13A; RpS7; RpS3A; RpL19; RpL40; RpL7A; hoip; RpP2; RpL35; RpS19; RpL13; RpL27; RpL23; RpL7; RpLP2; mRpL15; RpS27A; RpL7; sop; RpL34; CG6764; RpS3A; RpL26; RpL10Ab; RpL22; RpLP0; RpL36; RpS8; RpL32; RpL23A; RpS23; RpL18 | 1554; 2128; 3180; 5179; 5825; 6121; 6292; 6884; 7055; 7626; 11265; 11979; 12225 15319; 18009; 1475; 1883; 2168; 2746; 2960; 3314; 3949; 4087; 4111; 4464; 4651; 4759; 4863; 4897; 4918; 5219; 5271; 5502; 5920; 6090; 6764; 6779; 6846; 7283; 7434; 7490; 7622; 7808; 7939; 7977; 8415; 8615 | **General Transcription**; RNA polymerase II 215 KD subunit; Histone deacetylase; RNA polymerase II 140 KD subunit; Cdk9 (binds cycT); Histone H3.3A; Histone acetyltransferase; Transcription elongation regulator (binds Cdk9); Mediator complex; DNA binding, chromatin remodeling; Transcription elongation regulator; DNA topoisomerase and sigma DNA polymerase activity; RNA directed RNA polymerase; stimulates transcription elongation by RNA pol II CBP/p300, transcription; general RNA pol II transcription factor; **Ribosome proteins**; Ribosomal protein, structural constituent; Ribosomal protein, structural constituent; Ribosomal protein, structural constituent; Ribosomal protein, structural constituent; Ribosomal protein, structural constituent; Ribosomal protein, structural constituent; Ribosomal protein, structural constituent; Ribosomal protein, structural constituent; Ribosomal protein, structural constituent; Ribosomal protein, structural constituent; Ribosomal protein, structural constituent; Ribosomal protein, structural constituent; Ribosomal protein, structural constituent; Ribosomal protein, structural constituent; Ribosomal protein, structural constituent; Ribosomal protein, structural constituent; Ribosomal protein, structural constituent; Ribosomal protein, structural constituent; Ribosomal protein, structural constituent; Ribosomal protein, structural constituent; Ribosomal protein, structural constituent; Ribosomal protein, structural constituent; Ribosomal protein, structural constituent; Ribosomal protein, structural constituent; Ribosomal protein, structural constituent; Ribosomal protein, structural constituent; Ribosomal protein, structural constituent; Ribosomal protein, structural constituent; Ribosomal protein, structural constituent; Ribosomal protein, structural constituent; Ribosomal protein, structural constituent; Ribosomal protein, structural constituent |  |
| Table S1. Continued | | | |

| Name | CG | Function |
| --- | --- | --- |
| RpS18 \|; RpL37 \|; RpL24 \|; RpL34b \|; RpS26 \|; RpS6 \|; RpS12 \|; RpS4 \|; CG11386 \|; mRpS22 \|; RpL21 \|; RpS30 \|; RpL15 \| sbr; CG1810; CG2100; Pabp2; CG2807; CG3056; CG3605; Tis11; CG4119; l(2)35Df; Srp54; me31B; pAbp; SmB; Rox8; CG6015; CG6905; CG6937; CG6995; Hel25E; Rrp6; SmD3; tsu; CG8877; mago; CG10214; pUf68; CG14800; RnpS1; DebB; CG32344 | 8900 \|; 9091 \|; 9282 \|; 9354 \|; 10305 \|; 10944 \|; 11271 \|; 11276 \|; 11386 \|; 12261 \|; 12775 \|; 15697 \|; 17421 \| 1664; 1810; 2100; 2163; 2807; 3056; 3605; 4070; 4119; 4152; 4602; 4916; 5119; 5352; 5422; 6015; 6905; 6937; 6995; 7269; 7292; 8427; 8781; 8877; 9401; 10214; 12085; 14800; 16788; 16792; 32344 | Ribosomal protein, structural constituent; Ribosomal protein, structural constituent; Ribosomal protein, structural constituent; Ribosomal protein, structural constituent; Ribosomal protein, structural constituent; Ribosomal protein, structural constituent; Ribosomal protein, structural constituent; Ribosomal protein, structural constituent; Ribosomal protein, structural constituent; Ribosomal protein, structural constituent (mitochondrial); Ribosomal protein, structural constituent; Ribosomal protein, structural constituent; Ribosomal protein, structural constituent; **RNA processing**; mRNA nuclear export; mRNA capping, phosphatase; mRNA polyadenylation; Poly A binding protein, mRNA polyadenylation; pre-mRNA splicing factor; pre-mRNA splicing factor; pre-mRNA splicing factor; DNA/RNA binding, RNA catabolism, intracellular signaling; RNA binding domain; RNA DEAD box helicase; pre-mRNA splicing factor; Dead box helicase; Poly A binding protein, mRNA polyadenylation; pre-mRNA splicing factor; Poly A binding protein, mRNA splicing factor; pre-mRNA splicing factor; pre-mRNA splicing factor; RNA binding domain; RNA binding domain; RNA helicase, pre-mRNA splicing factor; rRNA metabolism, RNAse; nuclear mRNA splicing; Binds Mago, mRNA splicing and transport; pre-mRNA splicing factor; (binds tsunagi) part of exon junction complex, mRNA splicing & transport; 3'-5' riboexonuclease; pre-mRNA splicing factor; exoribonuclease; pre-mRNA splicing factor; pre-mRNA splicing factor; ATP dependent RNA helicase |

Table S1. Continued

| Name | CG | Function |
| --- | --- | --- |
| Rpt1; Pros45; Prosα7; CG2241; Prosβ2; Mov34; Pros28.1; Uch-L3; Rpt4; Rpn12; Pros35; Pros26.4; Rpn7; Pros54; Rpn1; Pros29; CG9475; CG9868; Rpn6; Tbp-1; Dox-A2; ProsMA5; Rpn2; Prosβ3; CG12000; Prosβ5; Rpt3; CG17331; Rpn11; Prosα6 | 1341; 1489; 1519; 2241; 3329; 3416; 3422; 3431; 3455; 4157; 4904; 5289; 5378; 7619; 7762; 9327; 9475; 9868; 10149; 10370; 10484; 10938; 11888; 11981; 12000; 12323; 16916; 17331; 18174; 18495 | Proteasome; (Proteasome regulatory particle; Proteasome regulatory particle; Proteasome core complex; Proteasome regulatory particle; Proteasome core complex; Proteasome regulatory particle; Proteasome core complex; Proteasome regulatory particle; Proteasome regulatory particle; Proteasome regulatory particle; Proteasome core complex; Proteasome regulatory particle; Proteasome regulatory particle; Proteasome regulatory particle; Proteasome regulatory particle; Proteasome core complex; Proteasome regulatory particle; Proteasome core complex; Proteasome regulatory particle; Proteasome regulatory particle; Proteasome regulatory particle; Proteasome core complex; Proteasome regulatory particle; Proteasome core complex; Proteasome core complex; Proteasome core complex; Proteasome regulatory particle; Proteasome core complex; Proteasome regulatory particle; Proteasome core complex) |
