# Supplementary material for: Identification of Drosophila Gene Products Required for Phagocytosis of Candida albicans
Source: PLoS Biol. 2005 Dec 20;4(1):e4. doi: 10.1371/journal.pbio.0040004 (PMC1310651; doi:10.1371/journal.pbio.0040004)
Supplement: Supplementary file 2 [file pbio.0040004.st002.doc]

Table S2. Measurement of the percentage of S2 cells phagocytosing after treatment with dsRNA against individual genes.

| Name | CG | Homolog | Function | Known phago. | C.a | E.c | Beads |
| --- | --- | --- | --- | --- | --- | --- | --- |
| Gel; γCop; comt; MED26; ras; Hr38; CkIα; CG2111; CG2158; MTA1-like; Rac1; Jra; CG2508; Parg; Tra1; CG2993; Traf1; SNF1A; colt; sob; Bap170; Vinc; Pxd; CG3494; Slh; blw; CG3638; CG3483; CG3891; Chi; Cop; Unc-76; Srp | 1106; 1528; 1618; 1793; 1799; 1864; 2028; 2111; 2158; 2244; 2248; 2275; 2508; 2864; 2905; 2993; 3048; 3051; 3057; 3242; 3274; 3299; 3477; 3494; 3539; 3612; 3638; 3483; 3891; 3924; 3948; 3981; 3992 | Gelsolin; gamma coat protein; NSF; No significant mammalian homology; IMPDH1; Orphan nuclear receptor NR4A2; caesin kinase 1 alpha; TRH specific aminopeptidase; nucleoporin 50/NPAP60/Nup50; Mta3; Rac1 (mammals); Jun (mammals); APC8; poly(ADP-ribose) glycohydrolase(Parg); TRRAP; Arrdc1 (arrestin domain containing 1); TRAF (mammals); AMP activated protein kinase; carnitine/acyl carnitine translocase; Odd skipped related 2A; No significant mammalian homology; Vinculin; Lactoperoxidase; Unknown function; sly1p; ATP synthase alpha subunit; tweety homol. 2 (ttyh2) weak homolog; isocitrate dehydrogenase 3 (NAD+) α; CBF-B; LIM domain binding protein; zeta coat protein; FEZ2 (also FEZ1); No significant mammalian homolog | Actin binding, actin depolymerization; COPI vessicle coat, retrograde transport golgi to ER; Intracellular protein transport, ATPase activity, NSF homolog; Transcription factor; IMP dehydrogenase, GMP synthesis; Hormone receptor transcription factor; Casein Kinase I alpha, protein signaling; Aminopeptidase; Nuclear protein import/export; Glucocorticoidreceptor like Dna binding domain, Zinc finger; JNK cascade signaling, cytoskeletal rearrangements; JNK cascade signaling, TF; APC8/CDC23; poly(ADP-ribose) glycohydrolase; transcription regulator, receptor signaling, ser/thr kinase; arrestins play role in endocytosis; JNK cascade signaling, Toll signaling, IAP1 interaction; G protein coupled receptor kinase, ST kinase; Carnitine/acyl carnitine carrier; RNA polymerase II transcription factor; DNA binding, brahma complex; Actin binding, cytoskeletal anchoring; Heme dependent peroxidase; Unknown; SNARE binding, exocytosis, intracellular protein transport; proton transporting ATP synthase, alpha subunit; Homolog to Tweety? Transmembrane protein; Isocitrate dehydrogenase; CCAAT-binding transcription factor; Lim domain binding protein, transcription factor; COPI vesicle coat; Axon cargo transport? (mammal FEZ2 interacts with PKC; glucocorticoid receptor like, GATA binding,transcription factor | YES; YES; YES; NO; NO; YES; NO; NO; NO; NO; YES; YES; NO; NO; NO; NO; NO; YES; NO; NO; NO; YES; NO; NO; NO; NO; NO; NO; NO; NO; YES; NO NO | 16; 22; 44; 30; 10; 16; 14; 38; 32; 42; 24; 14; 22; 30; 28; 34; 44; 32; 40; 12; 26; 36; 38; 44; 38; 10; 22; 28; 32; 32; 30; 30; 20 | 46; 26; 40; 34; 32; 44; 28; 68; 38; 48; 28; 42; 44; 38; 20; 56; 38; 46; 48; 30; 36; 40; 52; 26; 30; 40; 30; 36; 48; 36; 42; 34; 32 | 30; 11; 24; 33; 15; 14; 22; 29; 41; 35; 41; 31; 29; 48; 30; 28; 38; 34; 33; 32; 43; 48; 30; 47; 33; 15; 29; 48; 37; 26; 35; 41; 26 |

Table S2. Continued

| Name | CG | Homolog | Function | Known phago. | C.a | E.c | Beads |
| --- | --- | --- | --- | --- | --- | --- | --- |
| Smr; Act5C; svr; PNUTS; CG4136; Syx5; fzy; CG4328; smt3; SerT; CG4552; CG4615; SCAR; CG4780; cyp33; Pdh; Sra-1; atx2; Act88F; CG5198; Zn72D; Ip259; T-cp1; ken; CG5591; CG5599; Pp1-87B; CG5715; CG5720; CG5745; CG5790 | 4013; 4027; 4122; 4124; 4136; 4214; 4274; 4328; 4494; 4545; 4552; 4615; 4636; 4780; 4886; 4899; 4931; 5166; 5178; 5198; 5215; 5277; 5374; 5575; 5591; 5599; 5650; 5715; 5720; 5745; 5790 | NCoR1 (Small regions of homology); Actin; carboxypeptidase D; No significant mammalian homology; No significant mammalian homology; Syntaxin 5a (Stx5a); Fzy; LMX1B; Smt3; Na+ dependent serotonin transporter; Mammals: have homolog; monocyte-to-macrophage differentiation factor 2; SCAR; Golgi SNARE; Cyclophilin; alcohol dehydrogenase; FMR1 interacting protein; No significant mammalian homology; Actin; CD2 tail binding protein; ZFR; TIP1, Hussy 29, LNR42; TCP-1 alpha; Homology in Zinc finger only; MLL; dihydrolipoamide branched chain transacylase; PP1 gamma (catalytic); Meprin A beta subunit; NY-REN-37 antigen (weak homology); TBC1 domain family member 22B; Cdc7 | Transcription corepressor; Actin, cytoskeleton; metallocarboxypeptidase, processing of proteins that transit secretory pathway; Zinc finger domain, conserved with elongin A, and CRSP70; homeobox domain transcription factor; t-Snare,; APC component CDC20 (Targets to D box); glucocorticoid receptor like, homeobox transcription factor; Ubiquitin like protein; Serotonin transporter, extracellular transport; Rab-GAP domain, Rhodanese cell cycle control domain; Defense response?; Actin binding, Arp 2/3 regulator, KETTE/Sra1/Abi interactor; SNAP receptor activity, exocytosis, intra Golgi transport; RNA binding, Defense response; oxidoreductase, lipid metabolism; Rac1 associated (complex with kette,abi), regulates SCAR; actin organization; Actin, cytoskeleton; Defense response?; RNA binding, Zinc finger protein,; unknown; Group II chaperonin involved in correct Actin folding; Transcription factor, C2H2 zinc finger; transcription factor, hematopoesis?; dihydrolipoamide branched chain transacylase; Protein phosphatase 1; Meprin A activity, proteolysis Secreted protein; antigen in patients with renal cell carcinoma; GTPase activator activity (Rab GAP); Ser/Thr kinase activity | NO; YES; NO; NO; NO; YES; NO; NO; NO; YES; NO; NO; YES; YES; NO; YES; NO; NO; YES; NO; NO; NO; NO; NO; NO; NO; NO; NO; NO; NO; NO | 44; 28; 40; 36; 20; 36; 34; 44; 10; 34; 36; 38; 16; 42; 32; 44; 32; 32; 30; 26; 30; 22; 20; 40; 28; 38; 36; 36; 12; 30; 14 | 50; 34; 42; 44; 32; 18; 20; 34; 30; 40; 36; 32; 22; 38; 46; 32; 56; 32; 24; 58; 52; 48; 42; 34; 36; 50; 24; 26; 46; 40; 46 | 30; 32; 41; 40; 17; 38; 47; 44; 28; 37; 32; 37; 27; 32; 43; 33; 34; 37; 26; 21; 28; 28; 33; 42; 34; 41; 46; 37; 16; 25; 31 |
| Table S2. Continued | | | | | | | |

| Name | CG | Homolog | Function | Known phago. | C.a | E.c | Beads |
| --- | --- | --- | --- | --- | --- | --- | --- |
| Prx5037; Hem; CG5844; cact; CG5853; CG5861; par-6; brm; Arc-p20; CG6091; CG6191; CG6197; βCop; Nup62; CG6302; CG6359; CG6364; CG6479; CG6486; Bx; Ngp; inaC; Snap; CG6652; cenB1A; rhea; CG6842; gig; mts; Kuz; CG7149 | 5826; 5837; 5844; 5848; 5853; 5861; 5884; 5942; 5972; 6091; 6191; 6197; 6223; 6251; 6302; 6359; 6364; 6479; 6486; 6500; 6501; 6518; 6625; 6652; 6742; 6831; 6842; 6975; 7109; 7147; 7149 | Peroxiredoxin; Nap1 (Nck associated protein); No significant mammalian homology; I-kappa-B; ABC subfamily G member 4; seven transmembrane domain protein; Par-6; SNF2alpha, SMARCA4, HBRM; Arc20P; Mammals: have homologous protein; Cdk5 and Abl enzyme substrate 1 (CABLES); XPA binding protein 2 (XAB2); beta coat protein; Nup62; prefoldin 2; sorting nexin; uridine-cytidine kinase 2; Mammals: have homologous protein; PTS2 receptor; Homology in LIM domain only; Nucleolar GTPase; Protein kinase C beta; SNAP; No significant mammalian homology; CentB5 (mammals); ERGIC-53; Peptide deformylase; Tsc2/Tuberin; PP2 alpha or beta catalytic subunit; Desintegrin metalloprotease (MADM) or ADAM10 or kuzbanian; Ethanolaminephosphotransferase | defense response, oxygen & reactive oxygen metabolism; Binds Rac1 (Sra1, Abi complex) regulate SCAR; Fatty acid beta oxidation, enoyl CoA hydratase; NFkB cascade, transcription factor, defense; ABC transporter; Receptor activity; Protein binding, protein targeting, apical cell membrane; SWI/SNF related chromatin remodeling complex; Arp2/3 complex, actin binding, cytoskeletal rearrangement; Possible Cysteine protease; Cyclin like, human: links cAbl to Cdk5; transcription factor, DNA repair; COPI vesicle coat, protein transport; Nuclear pore protein; bind actin, tubulin and target to TCP-1 chaperonin; intracellular protein transport; Uridine kinase; Unknown function; peroxisome targeting signal-2 receptor activity; Glucocorticoid receptor like DNA binding domain; GTP binding, receptor binding, intracellular protein transport; Protein Kinase C, signaling; SNAP Intracellular protein transport; Unknown function; Arf GAP, GPCR signaling, cytoskeletal organization; actin binding, protein transport, defense response; peptide deformylase, protein modification; small GTPase regulatory/interacting activity, binds Tsc; Pp2A, attachment of Microtubule to kinetochore; metalloendopeptidase, Notch processing; diacylglycerol cholinephosphotransferase | NO; NO; NO; NO; NO; NO; NO; NO; YES; NO; NO; NO; YES; NO; NO; NO; NO; NO; NO; NO; NO; YES; YES; NO; NO; NO; NO; NO; NO; NO; NO | 34; 30; 36; 24; 24; 32; 16; 26; 6; 36; 26; 38; 40; 24; 28; 40; 32; 40; 30; 18; 36; 38; 44; 38; 26; 18; 26; 12; 26; 38; 44 | 30; 28; 56; 40; 20; 42; 38; 42; 22; 30; 44; 34; 28; 24; 46; 28; 16; 34; 30; 32; 18; 36; 20; 46; 24; 42; 30; 34; 40; 40; 24 | 30; 21; 39; 21; 25; 26; 19; 33; 30; 40; 34; 31; 34; 40; 35; 44; 42; 33; 36; 40; 38; 45; 40; 45; 33; 31; 34; 28; 43; 39; 41 |

Table S2. Continued

| Name | CG | Homolog | Function | Known phago. | C.a | E.c | Beads |
| --- | --- | --- | --- | --- | --- | --- | --- |
| CG7274; CG7275; Sox21a; CG7359; CG7362; CG7364; CG7365; CG7367; CG7368; Cka; Trn; Hn; Arp66B; CalpA; Mcr; CG7589; CG7593; CG7597; ATPsyn-γ; CG7633; sr; Fad2; axn; gt; αCop; eIF-1A; scb; ATPsyn-b; Garz; HLH106 | 7274; 7275; 7345; 7359; 7362; 7364; 7365; 7367; 7368; 7392; 7398; 7399; 7558; 7563; 7586; 7589; 7593; 7597; 7610; 7633; 7847; 7923; 7926; 7952; 7961; 8053; 8095; 8189; 8487; 8522 | ARID4b, Rbp1-like; Mammals: have homologous protein; Sox21 (weak homolog); Sec22; pyruvate kinase 3; TM9SF4, also EMP70; Phospholipase B; Triacylglycerol lipase; No significant mammalian homology outside Zinc finger; Striatin (mammals); Transportin 1/Karyopherin; phenylalanine hydroxylase; Arp2/3; Calpain 3; CD109/alpha2 macrobulin; glycine receptor alpha 3; Mammals: have homologous protein; Cdc 2-like protein kinase 5; ATP synthase gamma subunit; stomatin; Egr2; stearoyl CoA desaturase; Axin 1 or Axin 2 (conductin, axil); No significant mammalian homology; alpha coat protein; eIF-1A; various integrin alpha's; ATP synthase subunit b; GBF1, golgi specific brefeldin A resistance factor 1; SREBP-2 | Transcription factor,; WD repeat; HMG box transcription factor, transcriptional repressor, role in cell fate decisions; SNAP receptor, exocytosis, intracellular protein transport; pyruvate kinase, glycolysis; Endosome transmembrane protein; Phospholipase; triacylglycerol lipase, lipid metabolism; C2H2 Zinc finger; JNK cascade signaling; protein carrier, protein nucleus transport; phenylalanine 4 monooxygenase; Arp2/3 complex, actin binding, cytoskeletal rearrangement; Calcium, calmodulin binding cys protease; endopeptidase inhibitor, cytokine, signaling, defense response; GABA receptor, glycine gated ion channel, linked to microtubules and vesicle transport?; GCN5-related N acetyltransferase; Kinase domain is similar to Cdk5, N terminus no homology; ATP synthase complex, ATPase; integral membrane protein; transcription factor, C2H2 type; Stearoyl CoA 9-desaturase; Beta-catenin binding, wnt signaling; Transcriptional repressor; COPI vesicles, endocytosis; translation initiation; receptor activity/cell adhesion; ATP synthase complex; Arf guanyl nucleotide exchange factor; transcription factor, SREBP homolog | NO; NO; NO; NO; NO; NO; NO; YES; NO; NO; NO; NO; YES; YES; NO; NO; NO; NO; NO; NO; NO; NO; NO; NO; YES; NO; NO; NO; NO; NO | 44; 26; 42; 15; 24; 24; 42; 32; 38; 32; 32; 40; 24; 28; 32; 40; 32; 44; 44; 42; 38; 18; 16; 20; 18; 18; 44; 40; 44; 44 | 18; 42; 44; 60; 46; 50; 34; 32; 40; 38; 54; 22; 40; 32; 46; 34; 46; 46; 34; 46; 48; 32; 50; 46; 22; 64; 36; 44; 16; 44 | 40; 32; 44; 35; 31; 31; 37; 41; 45; 45; 41; 32; 24; 44; 41; 37; 30; 56; 29; 39; 42; 30; 28; 33; 25; 30; 58; 32; 13; 45 |
| Table S2. Continued | | | | | | | |

| Name | CG | Homolog | Function | Known phago. | C.a | E.c | Beads |
| --- | --- | --- | --- | --- | --- | --- | --- |
| Rac2; flfl; CG9449; CG9527; alien; Rab35; Int6; CG9737; Abi; CG9779; p16-ARC; CG10194; Nup98; S6k; CG10540; CG10542; CG10563; Sse; Caup; hook; Pat1; brat; CG10724; Tsp3A; CG10778; CG11132 | 8556; 9351; 9449; 9527; 9556; 9575; 9677; 9737; 9749; 9779; 9881; 10194; 10198; 10539; 10540; 10542; 10563; 10583; 10605; 10653; 10695; 10719; 10724; 10742; 10778; 11132 | Rac2; Mammals: have homologous protein; Prostatic acid phosphatase (PAP); Acyl-CoA oxidase, pristanoyl CoA oxidase; COP9 signalosome subunit 2; Rab35; eIF3 subunit 6, p48, INT-6; mannose-binding lectin associated serine protease 2 (Masp2); Abi1, hNap1; Vacuolar protein sorting 24 (Vps24); p16-ARC; similar to RP2 protein - testosterone regulated; Nup98; S6K ribosomal protein S6kinase; CAPZA1, F actin capping protein α 1; Ring finger protein 40, RBP95; GTF3C5, transcription factor IIIc63; No significant mammalian homology; IRX-6, iroquois homeodomain protein, (has homologous region); Hook3, hHK3; amyloid beta precursor protein binding protein and PAT1; TRIM3 tripartite motif protein3 & Lin41; WD repeat protein 1, Wdr1, Alix/Aip1; Mouse: tetraspanin 5, Tspan 5, human: tetraspan NET-4; Dehydrodolichyl diphosphate synthase isoform b; DNA methyltransferase 1 associated protein1 | JNK cascade signaling, cytoskeletal rearrangements; Rac protein signal transduction; Acid phosphatase; fatty acid beta oxidation, Acyl CoA dehydrogenase; COP9 signalosome (promotes cullin ubiquitin ligase); Small monomeric GTPase,; eIF3 complex, translation initiation (interacts with COP9); monophenol monooxygenase, serine protease; Interacts with Kette and Sra1 to regulate SCAR; SNF7 protein sorting and transport from endosome to vacuole/lysosome; Actin binding, Arp2/3 complex; Unknown; Nuclear pore protein; Binds Actin, In PI3K pathway; barbed end actin capping activity; structural constituent of cytoskeleton; Transcription factor; mitotic sister chromatid separation; homeodomain, iroquois complex; microtubule binding, endocytic trafficking; Microtubule motor; translational repressor; actin binding, actin depolymerization; cell-cell adhesion; acyltransferase, dilichol is glycosyl carrier lipid in synthesis of N linked glycoprotein and O linked oligosaccharides; transcriptional repressor | YES; NO; NO; NO; NO; NO; NO; YES; YES; NO; YES; NO; NO; NO; NO; NO; NO; NO; NO; NO; NO; NO; NO; NO; NO; NO | 34; 44; 38; 38; 28; 40; 40; 44; 30; 20; 40; 42; 34; 36; 32; 40; 38; 34; 44; 20; 36; 32; 42; 44; 36; 42 | 32; 50; 54; 38; 46; 34; 50; 36; 40; 40; 36; 52; 48; 32; 24; 44; 38; 34; 50; 34; 34; 46; 32; 36; 32; 32 | 42; 49; 50; 31; 45; 46; 55; 41; 50; 32; 51; 40; 42; 56; 54; 53; 54; 55; 46; 39; 36; 37; 41; 52; 41; 56 |

Table S2. Continued

| Name | CG | Homolog | Function | Known phago. | C.a | E.c | Beads |
| --- | --- | --- | --- | --- | --- | --- | --- |
| Tig; Pi3K68D; Ubi-p63E; CG11642; CG11652; Act42A; CG12071; CG12077; tth; polo; Cdc42; CHES-1-like; CG12750; CG12855; Cas; CG13284; Mnt; CG14606; Cop; CG16791; pnt; Pros; Pp2A-29B; CG17652; CG17754; CG30427; CG31064 | 11527; 11621; 11624; 11642; 11652; 12051; 12071; 12077; 12175; 12306; 12530; 12690; 12750; 12855; 13281; 13284; 13316; 14606; 14813; 16791; 17077; 17228; 17291; 17652; 17754; 30427; 31064 | no significant mammalian homology; PI3K; poly ubiquitin C; Translocation chain membrane associated protein 1 (TRAM1); OVCA1 and DPH2L; Actin; no significant mammalian homology; PIG C, phosphatidylinositol glycan class C; no significant mammalian homology; PLK1, polo-like kinase 1; Cdc42; No significant mammalian homology; Mammals: have homologous protein; Hermansky-Pudlak syndrome 1 protein; CSE1 like; Steroid dehydrogenase like; No significant mammalian homology; Solute carrier family 4 (SLC2A6); delta coat protein; No significant mammalian homology; Ets1; Prox1; PP2A regulatory domain, Pr65 alpha; mammal: have homologous protein; Kelch-like protein 5 (KLHL5); Male sterility domain containing 2; Rabip4 (mouse) RUN and FYVE domain-containing 2 (RUFY)(human) | ligand for αPS2βPS integrins; PI3Kinase; Ubiquitin; SRPdependent cotranslational membrane targeting; Dipthamide synthase, mammalian homolog no known function except as tumor suppressor; Actin, cytoskeleton; Zinc finger; glycosyl transferase (GPI membrane anchor biosynthesis); Transcription factor; S/T kinase, mitotic kinase, phosphorylates APC/C; Small GTPase, actin rearrangements, binds par6; Forkhead transcription factor; Unknown; Unknown; importin alpha export receptor; steroid dehydrogenase; Transcription factor (Myc, Max, Mad family); Hexose transporter; COPI vesicles, endocytosis; Unknwon; MAPKKK pathway, nuclear effector of EGFR signaling; positively regulates cycE promoter of glial cell, activates notch and antagonizes Dacapo; Protein phosphatase; Unknown; Actin binding protein; Oxidoreductase; Endocytosis, Rab 4 effector in mouse | NO; YES; YES; NO; NO; YES; NO; NO; NO; NO; YES; NO; NO; NO; NO; NO; NO; NO; YES; NO; NO; NO; NO; NO; NO; NO; NO | 32; 34; 40; 42; 40; 38; 24; 40; 34; 42; 44; 28; 44; 32; 24; 12; 40; 36; 42; 36; 44; 32; 38; 36; 40; 24; 28 | 54; 20; 24; 50; 30; 30; 52; 54; 42; 36; 46; 38; 60; 46; 26; 60; 30; 50; 20; 52; 38; 46; 40; 48; 40; 34; 40 | 25; 44; 33; 45; 50; 52; 42; 39; 38; 40; 47; 42; 46; 34; 38; 26; 36; 41; 41; 50; 45; 50; 36; 41; 35; 52; 30 |

Table S2. Continued

| Name | CG | Homolog | Function | Known phago. | C.a | E.c | Beads |
| --- | --- | --- | --- | --- | --- | --- | --- |
| CG31132; CG31345; Vha16-2; CG32465; CG32697; CG32776 | 31132; 31345; 32089; 32465; 32697; 32776 | bromo domain-containing protein disrupted in leukemia; Calcyphosine; ATPase V0 subunit c, lysosomal; Neuroligin; No significant mammalian homology; No significant mammalian homology | WD repeat; Ca2-binding; Hydrogen exporting ATPase; neurexin binding, receptor binding, defense response; Protein tyrosine phosphatase; Small GTPase | NO; NO; YES; NO; NO; NO | 24; 30; 16; 12; 30; 38 | 38; 54; 58; 38; 32; 44 | 30; 47; 39; 15; 35; 52 |
